# A Unique Case of Psychogenic Binocular Diplopia

**DOI:** 10.1155/crop/8555901

**Published:** 2026-09-03

**Authors:** Bryce Patrick Johnson

**Affiliations:** ^1^ Department of Ophthalmology, Baylor Scott & White, Temple, Texas, USA, baylorscottandwhite.com

## Abstract

Diplopia is a common chief complaint in the ophthalmology clinic, and it is important to differentiate diplopia requiring urgent work‐up versus benign forms of diplopia that can be managed conservatively. This case demonstrates a 39‐year‐old male who reported the ability to move each eye independently resulting in chronic binocular diplopia. He reported having an extensive work‐up done by an outside ophthalmologist that returned unremarkable. His glasses on presentation had a mild myopic correction in both eyes (OU), 1.50 prism diopters (PD) base up (BU) in the right eye (OD), and 0.50 PD BU in the left eye (OS), which helped intermittently—but not completely—resolve his diplopia. On presentation, his visual acuity (VA) was 20/20 OU with correction. He had a normal sensorimotor exam, including being orthotropic in primary gaze, having full motility OU, having normal saccades OU, and having no nystagmus OU. The patient voluntarily moved his eyes in a rhythmic sequence of convergence ➔ left gaze ➔ convergence ➔ right gaze ➔ convergence ➔ repeat. This sequence can initially appear to exhibit independent movement of each eye when done rapidly, but close examination can expose it as simple voluntary ductions and vergences that fall within the domain of normal ocular motility. With proper education, reassurance, and follow‐up, the patient’s subjective visual disturbances improved. Prompt recognition of this motility sequence can result in favorable visual improvements and avoidance of unnecessary work‐up.

## 1. Introduction

Diplopia is a common chief complaint that ophthalmologists often encounter. One study reported that there are over 800,000 visits to the ophthalmology clinic and 50,000 visits to the emergency department each year for complaints of double vision [1]. Such complaints are often distressing to patients and can cause uncertainty in clinicians while deciphering through the myriad possible etiologies. It is estimated that 16% of the emergency room encounters for diplopia will be due to potentially life‐threatening etiologies, although extensive work‐ups with neuroimaging are far more prevalent at nearly 60% of ED visits for diplopia [1, 2].

Monocular diplopia is comfortably handled by most ophthalmologists, as it is not neurologic in origin and is due to abnormalities of the refractive media (e.g., uncorrected astigmatism, dry eye) [3]. The approach to evaluating monocular diplopia is relatively simple, and common tools in the ophthalmology office, such as a pinhole occluder, can be used to prove the diplopia is due to an optical origin [4]. However, binocular diplopia can be much more complicated and can be a harbinger of sinister underlying neurologic causes [5, 6]. Whether due to the complexities of or time‐consuming nature of in‐clinic diplopia tests, there is often a low threshold for the general ophthalmologist to refer these cases to a neuro‐ophthalmologist or recommend extensive work‐ups to rule out emergent conditions, such as strokes or tumors.

Diplopia can be nonorganic in nature, but it is not a common manifestation of neuro‐ophthalmic psychogenic disease. When it does occur, it is usually monocular in nature and usually occurs in children [7–9]. Although psychogenic monocular diplopia is a known phenomenon, there are no reported cases of psychogenic *binocular* diplopia. The purpose of this case report is to make clinicians aware of this phenomenon and to help clinicians rapidly identify one specific etiology of psychogenic binocular diplopia, which can help reassure the patient and avoid unnecessary work‐ups.

## 2. Case Report

A 39‐year‐old male presented for chronic double vision that began more than 20 years prior to presentation after he reportedly discovered he could move each eye independently. He states that during grade school, he and a few classmates discovered this ability, although his diplopia persisted while the others’ resolved over time. Moving each eye independently was leading to misalignment and double vision with two completely separate objects appearing side‐by‐side. Although he could voluntarily manifest these independent ocular movements, they would also occur involuntarily. For instance, the patient reported that if he did not pay attention, one or both eyes could independently drift from ocular alignment inward or outward resulting in double vision. When he experienced ocular drifting substantial enough to cause double vision, he would volitionally bring both eyes back into ocular alignment, which resolved the diplopia. He states that constant focusing and glasses with prisms were necessary to align the eyes and eliminate the binocular diplopia. Despite these symptoms, he denied it significantly limited his daily activities. The patient did not have any other ocular history, although he notably had multiple mental health comorbidities.

The initial ophthalmic technician’s work‐up during the consultation notably documented (1) glasses with myopic and astigmatic correction OU as well as 1.5 PD BU OD and 0.50 PD BU OS and (2) “motility: abnormal OU”. The exam with the ophthalmologist demonstrated a normal sensorimotor exam, including being orthotropic in primary gaze without phorias or tropias, having full motility OU, having normal saccades OU, and having no nystagmus OU. The patient voluntarily moved his eyes in a rhythmic sequence of convergence ➔ left gaze ➔ convergence ➔ right gaze ➔ convergence ➔ repeat; this sequence repeatedly ended with forceful blinking and facial grimacing [Video 1]. The remainder of this patient’s 8‐point eye exam was unremarkable. His ocular motility was further investigated by confirming that he could not manifest voluntary vertical divergence. This limitation appeared to have been a new revelation to him, and he was surprised. Next, it was determined that the patient could not keep one eye in primary while abducting the contralateral eye. After determination that the patient’s ocular motility was normal, reassurance was provided to the patient that his symptoms will eventually improve, and he was prescribed an updated glasses prescription as well as artificial tears as needed. Given the patient was not significantly bothered by his symptoms, no referral to psychiatry was placed. At 6 months, the patient reported a decrease in his anxiety and an improvement in his symptoms.

## 3. Discussion

It is well known that many functional ophthalmologic findings and diagnoses exist, such as functional vision loss and psychogenic ophthalmologic movement disorders (POMDs) [9, 10]. However, the specific psychogenic disease affecting ocular motility described in this case report has not been presented before. We are calling the sequence of ocular movements described here “chameleon eyes” due to the *appearance* of disconjugate eye movements, for which chameleons are believed to have such an ability [11]. It is also fitting because humans *cannot* truly move their eyes independently voluntarily, exemplifying the cultural connotations of what it means to “be” or “act” like a chameleon (e.g., blending in to one’s surroundings and adopting others’ behaviors). It is possible that there are other variations of motility patterns that could fit under the category of chameleon eyes that have not been discovered yet.

In clinical scenarios where chameleon eyes are suspected, clinicians should follow similar steps to other POMDs [9]. The first step is to confirm that there is a normal sensorimotor exam without an organic disorder. In cases similar to the one presented here, several tests can be done clinically to discern true monocular independence from physiologic eye movements. The first and simplest is to interrupt the typical sequence by having the patient keep one eye in primary and attempt isolated abduction of the contralateral eye. The second hint is that the patient cannot demonstrate independent eye movements in the vertical plane. A third hint that the ocular movements are volitional and not pathologic is the presence of characteristic behaviors, such as fluttering of the eyelids, forceful blinking, or strained facial expressions [9, 12]. Finally, the clinician should determine whether the patient has an underlying psychiatric disease or is experiencing psychosocial stress and refer him or her to a psychiatrist for evaluation if the patient is significantly disturbed by the symptoms or impaired in daily activities. This referral is often not necessary if the patient is not significantly impaired, and treatment usually consists of education and reassurance [9, 10]. It is important to recognize this POMD to prevent unnecessary work‐up and provide rapid reassurance to the patient.

## Funding

No funding was received for this manuscript.

## Consent

All the patients allowed personal data processing and informed consent was obtained from all individual participants included in the study. Verbal consent was obtained during the initial consultation with the patient, and consent was re‐confirmed via telephone on 04/02/2026 prior to the submission of this case report.

## Conflicts of Interest

The author declares no conflicts of interest.

## Supporting information


**Supporting Information** Additional supporting information can be found online in the Supporting Information section Video 1: Chameleon eyes. The patient claims to be moving his eyes independently. Close observation reveals a rhythmic sequence of convergence ➔ left gaze ➔ convergence ➔ right gaze ➔ convergence ➔ repeat. Note how these movements only occur in the horizontal plane, how it can only be sustained for a short period of time, and how he grimaces and forcefully blinks at the termination of the movements.

## Data Availability

The data that support the findings of this study are available on request from the corresponding author. The data are not publicly available due to privacy or ethical restrictions.
